# Timing of adjuvant chemotherapy initiation and mortality among colon cancer patients at a safety-net health system

**DOI:** 10.1186/s12885-022-09688-w

**Published:** 2022-05-31

**Authors:** Yan Lu, Aaron W. Gehr, Rachel J. Meadows, Bassam Ghabach, Latha Neerukonda, Kalyani Narra, Rohit P. Ojha

**Affiliations:** 1 Center for Epidemiology & Healthcare Delivery Research, JPS Health Network, 1500 S. Main Street, Fort Worth, TX 76104 USA; 2 Department of Medical Education, TCU School of Medicine, 3430 Camp Bowie Blvd, Fort Worth, TX 76107 USA; 3 Oncology and Infusion Center, JPS Health Network, 1450 8th Ave, Fort Worth, TX 76104 USA

**Keywords:** Adjuvant chemotherapy, Timing, colon cancer, Quality of care, Prognosis, Mortality, Disease-free survival

## Abstract

**Background:**

Prior studies reported survival benefits from early initiation of adjuvant chemotherapy for stage III colon cancer, but this evidence was derived from studies that may be sensitive to time-related biases. Therefore, we aimed to estimate the effect of initiating adjuvant chemotherapy ≤8 or ≤ 12 weeks on overall and disease-free survival among stage III colon cancer patients using a study design that helps address time-related biases.

**Methods:**

We used institutional registry data from JPS Oncology and Infusion Center, a Comprehensive Community Cancer Program. Eligible patients were adults aged < 80 years, diagnosed with first primary stage III colon cancer between 2011 and 2017, and received surgical resection with curative intent. We emulated a target trial with sequential eligibility. We subsequently pooled the trials and estimated risk ratios (RRs) along with 95% confidence limits (CL) for all-cause mortality and recurrence or death at 5-years between initiators and non-initiators of adjuvant chemotherapy ≤8 or ≤ 12 weeks using pseudo-observations and a marginal structural model with stabilized inverse probability of treatment weights.

**Results:**

Our study population comprised 222 (for assessing initiation ≤8 weeks) and 310 (for assessing initiation ≤12 weeks) observations, of whom the majority were racial/ethnic minorities (64–65%), or uninsured with or without enrollment in our hospital-based medical assistance program (68–71%). Initiation of adjuvant chemotherapy ≤8 weeks of surgical resection did not improve overall survival (RR for all-cause mortality = 1.04, 95% CL: 0.57, 1.92) or disease-free survival (RR for recurrence or death = 1.07, 95% CL: 0.61, 1.88). The results were similar for initiation of adjuvant chemotherapy ≤12 weeks of surgical resection.

**Conclusions:**

Our results suggest that the overall and disease-free survival benefits of initiating adjuvant chemotherapy ≤8 or ≤ 12 weeks of surgical resection may be overestimated in prior studies, which may be attributable to time-related biases. Nevertheless, our estimates were imprecise and differences in population characteristics are an alternate explanation. Additional studies that address time-related biases are needed to clarify our findings.

**Supplementary Information:**

The online version contains supplementary material available at 10.1186/s12885-022-09688-w.

## Introduction

Adjuvant chemotherapy following surgical resection of colon cancer is intended to eliminate micrometastatic disease and increase overall and disease-free survival [1]. Evidence from randomized controlled trials (RCTs) established that adjuvant chemotherapy increased survival among stage III colon cancer patients, [2–4] and thus adjuvant chemotherapy for stage III colon cancer is standard of care. In addition, evidence suggests that early initiation of adjuvant chemotherapy may improve survival among colon cancer patients, [5–8] and timing of adjuvant chemotherapy initiation is included in quality of care guidelines [9, 10]. Nevertheless, the evidence for timing of adjuvant chemotherapy was derived from observational studies, which may be sensitive to time-related biases [11, 12].

In contrast to RCTs, where the design ensures alignment of key elements such as treatment allocation, eligibility, and follow-up at a common starting point (i.e., time zero) to minimize time-related biases, this alignment requires additional methodologic considerations in observational studies [11, 12]. For example, prior studies [5–8] included comparisons of survival between patients who initiated adjuvant chemotherapy ≤8 weeks and patients who initiated adjuvant chemotherapy > 8 weeks following surgical resection. Such comparisons are problematic because patients must survive a specified duration before initiating treatment, and analyses that do not properly account for this duration will incur immortal time bias [11, 13–17]. Several studies have illustrated severe overestimation of treatment effects on survival in observational studies of cancer patients, which was attributable to immortal time bias [18–20]. Immortal time bias has not been addressed in the context of timing of adjuvant chemotherapy initiation for colon cancer survival and could have implications for current quality of care guidelines. Therefore, we aimed to estimate the effect of initiating adjuvant chemotherapy for stage III colon cancer within an interval of interest (8 or 12 weeks) following surgical resection on survival using a study design that helps align time zero and reduce immortal time bias.

## Methods

### Study population

We used institutional registry data from JPS Oncology and Infusion Center (JPS), an accredited Comprehensive Community Cancer Program. The center is part of an urban safety-net health system, which is the primary source of care for socioeconomically marginalized populations in Tarrant County, TX. Eligible patients were diagnosed with first primary stage III colon cancer between 2011 and 2017, aged 18–79 years at cancer diagnosis, received surgical resection with curative intent, and received at least part of the first course treatment at JPS. We excluded patients for whom adjuvant chemotherapy was contraindicated.

### Variables

Our primary outcome of interest was 5-year overall survival (i.e., complement of all-cause mortality) and secondary outcome of interest was 5-year disease-free survival (i.e., complement of recurrence or mortality [21]). These outcomes were selected because adjuvant chemotherapy is intended to improve overall and disease-free survival [1]. In addition, the American Society of Clinical Oncology statement about clinically meaningful outcomes defines overall survival as the primary outcome of interest [22]. Our exposure (intervention) of interest was initiation of adjuvant chemotherapy within 8 or 12 weeks of surgical resection (i.e., initiators vs. non-initiators within 8 weeks in one analysis and initiators vs. non-initiators within 12 weeks in a separate analysis). We also extracted baseline information from the registry including age at diagnosis, sex, race/ethnicity, insurance coverage, marital status, comorbidities, body mass index, tumor grade, and surgical procedure.

### Data analysis

We emulated a sequence of observational “trials,” [11, 23–25] where study eligibility criteria were applied and intervention status was defined within a sequence of trials based on 2-week intervals through 8 or 12 weeks from surgical resection. One exception was that the interval for the first trial was 4 weeks because no one initiated adjuvant chemotherapy within 2 weeks of surgical resection. Baseline (i.e., time zero) for the first trial was the date of surgical resection. For the first trial, patients were classified as initiators if adjuvant chemotherapy was initiated ≤4 weeks (i.e., 28 days) of surgical resection, and as non-initiators if adjuvant chemotherapy was not initiated ≤4 weeks. We applied the eligibility criteria and initiator definition for sequential 2-week intervals, where time zero for each trial was the beginning of each interval. Consequently, patients could have been eligible for up to three trials for evaluating initiation ≤8 weeks and five trials for evaluating initiation ≤12 weeks of surgical resection but were no longer eligible for subsequent trials if adjuvant chemotherapy was initiated, the patient died, had recurrence (for disease-free survival), or were lost to follow-up in a previous trial.

We subsequently pooled data from all three (for evaluating initiation ≤8 weeks of surgical resection) or five trials (for evaluating initiation ≤12 weeks of surgical resection), which allowed for reducing variance [11, 23–25]. We fit a logistic regression model to compute stabilized inverse probability of treatment weights (IPTW) [26] for adjuvant chemotherapy initiation. Stabilized IPTW were based on a minimal sufficient set of covariates to reduce confounding bias identified using the back-door criterion in a directed acyclic graph of dependency assumptions [27–29] between adjuvant chemotherapy initiation and mortality or recurrence. The minimal sufficient set of covariates included baseline measurements of age, sex (male or female), race/ethnicity (non-Hispanic White, non-Hispanic Black, Hispanic, or non-Hispanic other), insurance coverage (uninsured without JPS Connection [a hospital-based medical assistance program for eligible individuals without insurance], uninsured with JPS Connection, or commercial/public insurance), marital status (single/never married, married, or divorced/separated/widowed), comorbidities classified by the National Cancer Institute [30] (0 or > 0), body mass index (BMI; BMI < 25, 25 ≤ BMI < 30, or BMI ≥ 30), tumor grade (well/moderately differentiated or poorly differentiated/undifferentiated), and surgical procedure (partial colectomy/segmental resection or hemicolectomy/subtotal/total colectomy). The standardized mean differences for covariates between initiators and non-initiators of adjuvant chemotherapy did not suggest meaningful imbalance after weighting except for one category of marital status in the 8-week analysis (Supplementary Table S1 and S2) [31].

We adjusted Kaplan-Meier estimators using stabilized IPTW [32] to generate marginal overall and disease-free survival for initiation and no initiation of adjuvant chemotherapy ≤8 or ≤ 12 weeks of surgical resection. For 5-year overall survival, patients were followed from date of surgical resection until death, loss to follow-up, or end of study, whichever occurred first. For 5-year disease-free survival, patients were followed from date of surgical resection until recurrence, death, loss to follow-up, or end of study, whichever occurred first. We also applied these weights in generalized linear models with pseudo-observations [33, 34] to construct marginal structural models [35] for estimating risk ratios (RR) for all-cause mortality and recurrence or death at 5 years comparing initiators and non-initiators of adjuvant chemotherapy ≤8 or ≤ 12 weeks of surgical resection. We estimated 95% confidence limits (CL) for RRs using clustered standard errors to account for repeated eligibility. Unlike Cox proportional hazard regression, the pseudo-observation approach does not require the proportional hazards assumption and allows estimating effect measures other than the hazard ratio, which is widely misinterpreted, has built-in selection bias, and no causal interpretation [36–40]. We estimated RR to provide a direct comparison of risk, which is easier to interpret than hazard [36, 38, 40].

## Results

Our analyses were based on 222 evaluable observations after pooling 3 sequential trials for evaluating initiation ≤8 weeks and 310 evaluable observations after pooling 5 sequential trials for evaluating initiation ≤12 weeks of surgical resection. Table 1 summarizes the distribution of the baseline characteristics of these observations. The median age of the study population was 56 years (interquartile range [IQR]: 50–61), and the majority were female (56–57%), racial/ethnic minorities (64–65%), or uninsured with or without enrollment in our hospital-based medical assistance program (68–71%). Median time from diagnosis to surgical resection was 4 days (IQR: 1–28). Median time from surgical resection to adjuvant chemotherapy was 56 days (IQR: 46–78) for observations that initiated adjuvant chemotherapy in the 8-week analysis and 69 days (IQR: 51–86) for observations that initiated adjuvant chemotherapy in the 12-week analysis. For the 8-week analysis, 28% did not initiate adjuvant chemotherapy at any time, and for the 12-week analysis 34% did not initiate adjuvant chemotherapy at any time. The most common reason for not initiating adjuvant chemotherapy was patient refusal (71%). FOLFOX (including modified FOLFOX) was the most common adjuvant chemotherapy regimen (71–73%).

**Table 1:** Characteristics of study populations with stage III colon cancer eligible for adjuvant chemotherapy following surgical resection

Characteristics	Study population for adjuvant chemotherapy initiation ≤ 8 weeks of surgical resection (***n*** = 222) n (%)	Study population for adjuvant chemotherapy initiation ≤ 12 weeks of surgical resection (***n*** = 310) n (%)
**Age (years)**		
**Median (Interquartile range)**	56 (50–61)	56 (50–61)
**Sex**		
**Female**	126 (57)	175 (56)
**Male**	96 (43)	135 (44)
**Race/Ethnicity**		
**Non-Hispanic White**	77 (35)	112 (36)
**Non-Hispanic Black**	67 (30)	87 (28)
**Hispanic**	57 (26)	87 (28)
**Non-Hispanic other**	21 (9.5)	24 (7.7)
**Insurance coverage**		
**Uninsured without hospital-based medical assistance program**	50 (23)	73 (24)
**Uninsured with hospital-based medical assistance program**	107 (48)	137 (44)
**Insured**^**a**^	65 (29)	100 (32)
**Marital status**		
**Single**	89 (40)	124 (40)
**Married**	70 (32)	99 (32)
**Divorced/Separated/Widowed**	63 (28)	87 (28)
**Body Mass Index (BMI)**		
**BMI < 25**	56 (25)	78 (25)
**25 ≤ BMI < 30**	59 (27)	87 (28)
**BMI ≥ 30**	107 (48)	145 (47)
**NCI comorbidity index**		
**0**	168 (76)	235 (76)
**> 0**	54 (24)	75 (24)
**Tumor grade**		
**Well/Moderately differentiated**	191 (86)	265 (85)
**Poorly differentiated/Undifferentiated**	31 (14)	45 (15)
**Surgery procedure**		
**Partial colectomy/Segmental resection**	106 (48)	143 (46)
**Subtotal/Hemicolectomy/Total colectomy**	116 (52)	167 (54)
**Timing of adjuvant chemotherapy initiation**		
**Initiation ≤ 8 weeks of surgical resection**	81 (36)	N/A
**No initiation ≤ 8 weeks of surgical resection**	141 (64)	N/A
**Initiation ≤ 12 weeks of surgical resection**	N/A	150 (48)
**No initiation ≤ 12 weeks of surgical resection**	N/A	160 (52)

^a^Commercial or public insurance

**Fig. 1:**
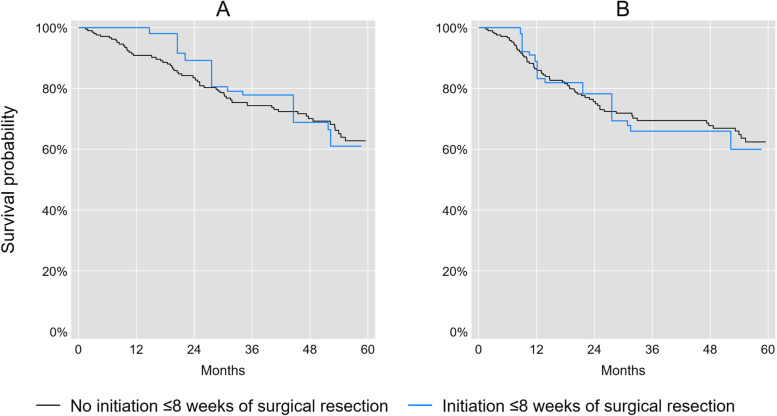
Marginal survival curves^a^ for initiation or no initiation of adjuvant chemotherapy ≤8 weeks of surgical resection for stage III colon cancer patients. **A** Overall survival. **B** Disease-free survival. (Adjusted for age, sex, race/ethnicity, insurance status, marital status, National Cancer Institue comorbidity index, body mass index, tumor grade, and surgical procedures)

**Fig. 2:**
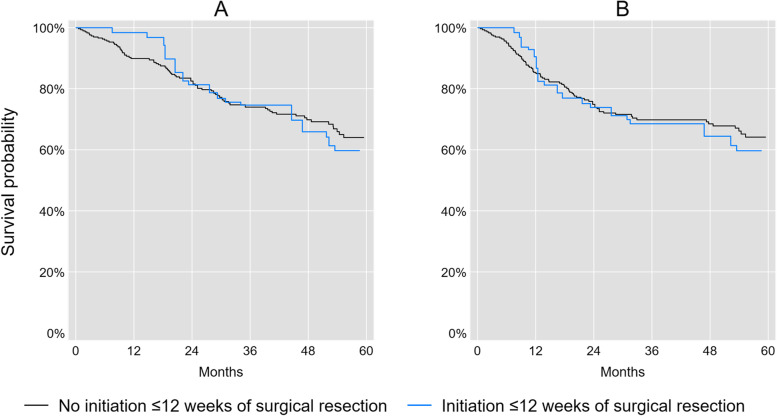
Marginal survival curves^a^ for initiation or no initiation of adjuvant chemotherapy ≤12 weeks of surgical resection for stage III colon cancer patients. **A** Overall survival. **B** Disease-free survival. (Adjusted for age, sex, race/ethnicity, insurance status, marital status, National Cancer Institue comorbidity index, body mass index, tumor grade, and surgical procedures)

We observed 62 deaths and 57 recurrences within 5 years of surgical resection among observations in the 8-week analysis, and 84 deaths and 79 recurrences among observations in the 12-week analysis. Figures 1 and 2 illustrate the marginal overall and disease-free survival curves for initiators and non-initiators of adjuvant chemotherapy ≤8 or ≤ 12 weeks of surgical resection. The crude risk of all-cause mortality was 17% lower (RR = 0.83; 95% CL: 0.46, 1.51) and the crude risk of recurrence or mortality was 7% lower at 5 years (RR = 0.93; 95% CL: 0.55, 1.57) for patients who initiated adjuvant chemotherapy ≤8 weeks compared with patients who did not initiate adjuvant chemotherapy ≤8 weeks of surgical resection. The crude risk of all-cause mortality was 14% higher (RR = 1.14; 95% CL: 0.77, 1.68) and the crude risk of recurrence or mortality was 22% higher at 5 years (RR = 1.22; 95% CL: 0.85, 1.76) for patients who initiated adjuvant chemotherapy ≤12 weeks compared with patients who did not initiate adjuvant chemotherapy ≤12 weeks of surgical resection.

The adjusted risk (i.e., after weighting) of 5-year all-cause mortality was 4% higher for patients who initiated adjuvant chemotherapy ≤8 weeks compared with patients who did not initiate adjuvant chemotherapy ≤8 weeks of surgical resection, but our data were compatible with 43% lower risk or 92% higher risk of mortality (RR = 1.04, 95% CL: 0.57, 1.92). The adjusted risk of recurrence or mortality at 5 years was 7% higher for patients who initiated adjuvant chemotherapy ≤8 weeks compared with patients who did not initiate adjuvant chemotherapy ≤8 weeks of surgical resection, but our data were compatible with 39% lower risk or 88% higher risk of recurrence or mortality (RR = 1.07, 95% CL: 0.61, 1.88). The results were similar for initiation of adjuvant chemotherapy ≤12 weeks of surgical resection (Table 2*).*

**Table 2:** Risk ratios (RRs) for all-cause mortality and recurrence or mortality at 5-years between initiators and non-initiators of adjuvant chemotherapy ≤8 or ≤ 12 weeks of surgical resection for stage III colon cancer patients

	Crude RR (95% CL^**a**^)	Adjusted RR (95% CL^**a**^)
**5-year mortality**		
**Initiation ≤8 weeks**	0.83 (0.46, 1.51)	1.04 (0.57, 1.92)
**Initiation ≤12 weeks**	1.14 (0.77, 1.68)	1.11 (0.74, 1.67)
**5-year recurrence or death**		
**Initiation ≤8 weeks**	0.93 (0.55, 1.57)	1.07 (0.61, 1.88)
**Initiation ≤12 weeks**	1.22 (0.85, 1.76)	1.12 (0.76, 1.67)

^a^*CL* Confidence limits

## Discussion

Our analysis aimed to align time zero and reduce immortal time bias, which were sources of error in prior studies about timing of adjuvant chemotherapy initiation for stage III colon cancer patients < 80 years. Our results suggest that the overall and disease-free survival benefits of initiating adjuvant chemotherapy ≤8 or ≤ 12 weeks of surgical resection may be overestimated in prior studies. Nevertheless, our data were compatible with either meaningful benefit or harm of initiation within specified intervals. Imprecision and potential sources of error require further consideration when interpreting our results.

Imprecision in survival analysis is a function of number of events (i.e., death or recurrence in our analysis) and person-time. A larger sample size or longer follow-up (assuming more events) could provide more precise estimates, but our sample size was limited to available data. Precision is certainly important but only addresses random error. Quantification of effects with reduced systematic error (i.e., mitigated biases) is also critical. Our analysis prioritized mitigating key biases in prior studies that could mislead interpretation. Consequently, our estimates may be useful despite imprecision, [41, 42] particularly if estimates from multiple studies with similar approaches are summarized in a meta-analysis to improve precision [41].

As with any observational study, our estimates may be sensitive to violations of exchangeability [43, 44] (i.e., unmeasured confounding or selection bias). For example, data were unavailable to allow adjustment for frailty at diagnosis. Nevertheless, unmeasured confounding by frailty would create bias away from the null because we would expect an inverse relation between frailty and initiation of adjuvant chemotherapy ≤8 or ≤ 12 weeks of surgical resection (i.e., initiation may require additional time for frail patients) and frailty would increase mortality risk. Adjustment would thus move the estimate further toward the null [45]. In addition, the crude and adjusted 95% confidence limits for risk ratios were largely overlapping despite adjustment for multiple covariates, particularly for the more stable 12-week estimates. The lack of notable differences in crude and adjusted estimates suggests that confounding may not be as prominent of a concern once time zero is aligned, which is a phenomenon observed in prior studies that explored the effects of misaligned time zero [23, 46]. Lastly, survival could be affected by adherence to adjuvant chemotherapy [47, 48], but addressing adherence would change the question of interest and require a different study design [49]. Our study was designed to address the effect of initiating adjuvant chemotherapy, which is the question of interest relevant to quality of care guidelines and the basis of prior studies.

Our findings differ from prior studies [50–53], in which point estimates suggested 25–55% lower mortality hazards for initiation of adjuvant chemotherapy ≤8 weeks after surgical resection compared with later initiation or no initiation among colon cancer patients. Time-related biases [11, 12, 14, 15, 54] from misaligned start of eligibility, time of treatment assignment, and start of follow-up are a key consideration for effect heterogeneity between our study and prior studies. For example, immortal time bias is a concern in prior studies [50–53, 55] because follow-up time was measured from surgical resection, but adjuvant chemotherapy was initiated after follow-up time began. The consequence is misclassified person-time, where the time between start of follow-up and treatment initiation is considered “immortal.” Several studies have reported substantial bias away from the null because of immortal time, [19, 20, 25, 56] and this bias can be more severe than unmeasured confounding [23, 46]. We used analytic methods to mitigate immortal time bias, [11, 57] which may partly explain why our point estimates are closer to the null than prior studies. In addition, prior studies [50–52] excluded patients who were eligible for but did not initiate adjuvant chemotherapy, which incurs selection bias [58]. Lastly, effect heterogeneity across studies may be related to clinical setting and population characteristics. For example, our study was conducted in a cancer center that provides care for socioeconomically marginalized populations. Our estimates may be closer to the null if adverse effects of social determinants of health override benefits of earlier treatment initiation [59, 60]. Consequently, our results may generalize to other safety-net settings but not necessarily academic cancer centers.

In summary, assuming no substantial effect of biases, our results suggest that initiating adjuvant chemotherapy ≤8 or ≤ 12 weeks of surgical resection for stage III colon cancer patients < 80 years may not be as beneficial as reported in prior studies. Nevertheless, our results were imprecise and require confirmation. Future studies that also address time-related biases and have larger samples or longer follow-up may provide greater precision. Alternatively, estimates from multiple similar studies may be combined in a meta-analysis to improve precision. Such evidence could be valuable considering that cancer care delivery organizations dedicate considerable resources to meet guidelines for timely care, but some guidelines may not be optimized for meaningful outcomes or for certain settings.

## Supplementary Information

**Additional file 1: **Supplementary Table S1, S2.

## Data Availability

The data analyzed for the current study are available on reasonable request to the corresponding author and review by the JPS Health Network External Data Governance Committee (research@jpshealth.org).

## Abbreviations

RR: Risk ratio
CL: Confidence limits
RCT: Randomized controlled trials
BMI: Body mass index
IPTW: Inverse probability of treatment weights
IQR: Interquartile range
