# Supplementary material for: Timing of adjuvant chemotherapy initiation and mortality among colon cancer patients at a safety-net health system
Source: BMC Cancer. 2022 May 31;22:593. doi: 10.1186/s12885-022-09688-w (PMC9158363; doi:10.1186/s12885-022-09688-w)
Supplement: Supplementary file 1 — Additional file 1: Supplementary Table S1, S2. [file 12885_2022_9688_MOESM1_ESM.docx]

**Supplementary Table S1 Standardized mean differences (SMD) for covariates between initiation and no initiation of adjuvant chemotherapy ≤8 weeks of surgery among socioeconomically disadvantaged patients with stage III colon cancer**

|  | Before weighting | | | After weighting | | |
| --- | --- | --- | --- | --- | --- | --- |
|  | Initiation ≤8 weeks of surgery  Mean (SD^a^) | No initiation ≤8 weeks of surgery  Mean (SD^a^) | SMD | Initiation ≤8 weeks of surgery  Mean (SD^a^) | No initiation ≤8 weeks of surgery  Mean (SD^a^) | SMD |
|  |  |  |  |  |  |  |
| Age (years) | 56 (11) | 52 (11) | 0.365 | 55 (11) | 54 (11) | 0.080 |
| Sex |  |  |  |  |  |  |
| Female | 0.56 (0.5) | 0.59 (0.5) | -0.049 | 0.57 (0.5) | 0.63 (0.49) | -0.123 |
| Male | 0.44 (0.5) | 0.41 (0.5) | 0.049 | 0.43 (0.5) | 0.37 (0.49) | 0.123 |
| Race/Ethnicity |  |  |  |  |  |  |
| Non-Hispanic White | 0.36 (0.48) | 0.29 (0.46) | 0.132 | 0.35 (0.48) | 0.31 (0.47) | 0.069 |
| Non-Hispanic Black | 0.29 (0.45) | 0.38 (0.49) | -0.201 | 0.3 (0.46) | 0.32 (0.47) | -0.045 |
| Hispanic or non-Hispanic other | 0.36 (0.48) | 0.32 (0.47) | 0.069 | 0.35 (0.48) | 0.36 (0.49) | -0.025 |
| Insurance |  |  |  |  |  |  |
| Uninsured without hospital-based  medical assistance program | 0.23 (0.42) | 0.18 (0.39) | 0.142 | 0.22 (0.42) | 0.2 (0.41) | 0.054 |
| Uninsured with hospital-based  medical assistance program | 0.45 (0.5) | 0.68 (0.47) | -0.472 | 0.48 (0.5) | 0.55 (0.5) | -0.134 |
| Insured^b^ | 0.32 (0.47) | 0.15 (0.36) | 0.413 | 0.29 (0.46) | 0.25 (0.44) | 0.101 |
| Marital status |  |  |  |  |  |  |
| Single/Unmarried | 0.39 (0.49) | 0.44 (0.5) | -0.096 | 0.4 (0.49) | 0.33 (0.48) | 0.133 |
| Married | 0.32 (0.47) | 0.26 (0.45) | 0.130 | 0.31 (0.47) | 0.29 (0.46) | 0.051 |
| Divorced/Separated/Widowed | 0.28 (0.45) | 0.29 (0.46) | -0.027 | 0.29 (0.45) | 0.38 (0.49) | -0.186 |
| Body Mass Index (BMI) |  |  |  |  |  |  |
| BMI<25 | 0.25 (0.43) | 0.26 (0.45) | -0.033 | 0.25 (0.44) | 0.21 (0.41) | 0.101 |
| 25≤BMI<30 | 0.28 (0.45) | 0.18 (0.39) | 0.251 | 0.27 (0.44) | 0.3 (0.46) | -0.068 |
| BMI≥30 | 0.47 (0.5) | 0.56 (0.5) | -0.181 | 0.48 (0.5) | 0.49 (0.51) | -0.024 |
| NCI comorbidity index |  |  |  |  |  |  |
| 0 | 0.76 (0.43) | 0.74 (0.45) | 0.058 | 0.76 (0.43) | 0.75 (0.44) | 0.016 |
| >0 | 0.24 (0.43) | 0.26 (0.45) | -0.058 | 0.24 (0.43) | 0.25 (0.44) | -0.016 |
| Tumor grade |  |  |  |  |  |  |
| Well/Moderately differentiated | 0.46 (0.5) | 0.59 (0.5) | -0.262 | 0.48 (0.5) | 0.47 (0.51) | 0.016 |
| Poorly differentiated/     Undifferentiated | 0.54 (0.5) | 0.41 (0.5) | 0.262 | 0.52 (0.5) | 0.53 (0.51) | -0.016 |
| Surgery procedure |  |  |  |  |  |  |
| Partial colectomy/     Segmental resection | 0.86 (0.35) | 0.88 (0.33) | -0.076 | 0.86 (0.35) | 0.9 (0.3) | -0.129 |
| Subtotal/Hemicolectomy/     Total colectomy | 0.14 (0.35) | 0.12 (0.33) | 0.076 | 0.14 (0.35) | 0.1 (0.3) | 0.129 |

^a^SD: Standard deviation

^b^ Commercial or public insurance

**Supplementary Table S2 Standardized mean differences (SMD) for covariates between initiation and no initiation of adjuvant chemotherapy ≤12 weeks of surgery among socioeconomically disadvantaged patients with stage III colon cancer**

|  | Before weighting | | | After weighting | | |
| --- | --- | --- | --- | --- | --- | --- |
|  | Initiation ≤12 weeks of surgery  Mean (SD^a^) | No initiation ≤12 weeks of surgery  Mean (SD^a^) | SMD | Initiation ≤12 weeks of surgery  Mean (SD^a^) | No initiation ≤12 weeks of surgery  Mean (SD^a^) | SMD |
|  |  |  |  |  |  |  |
| Age (years) | 56 (10) | 53 (10) | 0.336 | 56 (11) | 55 (10) | 0.070 |
| Sex |  |  |  |  |  |  |
| Female | 0.57 (0.5) | 0.55 (0.5) | 0.032 | 0.57 (0.5) | 0.59 (0.5) | -0.040 |
| Male | 0.43 (0.5) | 0.45 (0.5) | -0.032 | 0.43 (0.5) | 0.41 (0.5) | 0.040 |
| Race/Ethnicity |  |  |  |  |  |  |
| Non-Hispanic White | 0.38 (0.49) | 0.29 (0.46) | 0.191 | 0.36 (0.48) | 0.33 (0.47) | 0.070 |
| Non-Hispanic Black | 0.27 (0.44) | 0.35 (0.48) | -0.170 | 0.28 (0.45) | 0.29 (0.46) | -0.023 |
| Hispanic or non-Hispanic other | 0.36 (0.48) | 0.37 (0.49) | -0.023 | 0.36 (0.48) | 0.38 (0.49) | -0.047 |
| Insurance |  |  |  |  |  |  |
| Uninsured without hospital-based  medical assistance program | 0.25 (0.44) | 0.14 (0.35) | 0.277 | 0.23 (0.42) | 0.21 (0.41) | 0.066 |
| Uninsured with hospital-based  medical assistance program | 0.41 (0.49) | 0.63 (0.49) | -0.463 | 0.44 (0.5) | 0.47 (0.5) | -0.056 |
| Insured^a^ | 0.34 (0.47) | 0.22 (0.42) | 0.259 | 0.32 (0.47) | 0.32 (0.47) | 0.001 |
| Marital status |  |  |  |  |  |  |
| Single/Unmarried | 0.4 (0.49) | 0.39 (0.49) | 0.030 | 0.4 (0.49) | 0.35 (0.48) | 0.104 |
| Married | 0.33 (0.47) | 0.27 (0.45) | 0.140 | 0.32 (0.47) | 0.34 (0.48) | -0.043 |
| Divorced/Separated/Widowed | 0.27 (0.44) | 0.35 (0.48) | -0.170 | 0.28 (0.45) | 0.31 (0.47) | -0.066 |
| Body Mass Index (BMI) |  |  |  |  |  |  |
| BMI<25 | 0.25 (0.43) | 0.27 (0.45) | -0.037 | 0.25 (0.43) | 0.23 (0.43) | 0.037 |
| 25≤BMI<30 | 0.3 (0.46) | 0.2 (0.41) | 0.210 | 0.28 (0.45) | 0.27 (0.45) | 0.017 |
| BMI≥30 | 0.46 (0.5) | 0.53 (0.5) | -0.149 | 0.47 (0.5) | 0.49 (0.51) | -0.047 |
| NCI comorbidity index |  |  |  |  |  |  |
| 0 | 0.76 (0.43) | 0.76 (0.43) | 0.008 | 0.76 (0.43) | 0.78 (0.42) | -0.038 |
| >0 | 0.24 (0.43) | 0.24 (0.43) | -0.008 | 0.24 (0.43) | 0.22 (0.42) | 0.038 |
| Tumor grade |  |  |  |  |  |  |
| Well/Moderately differentiated | 0.44 (0.5) | 0.55 (0.5) | -0.213 | 0.46 (0.5) | 0.46 (0.5) | 0.010 |
| Poorly differentiated/     Undifferentiated | 0.56 (0.5) | 0.45 (0.5) | 0.213 | 0.54 (0.5) | 0.54 (0.5) | -0.010 |
| Surgery procedure |  |  |  |  |  |  |
| Partial colectomy/     Segmental resection | 0.85 (0.36) | 0.9 (0.31) | -0.153 | 0.86 (0.35) | 0.88 (0.33) | -0.060 |
| Subtotal/Hemicolectomy/     Total colectomy | 0.15 (0.36) | 0.1 (0.31) | 0.153 | 0.14 (0.35) | 0.12 (0.33) | 0.060 |

^a^SD: Standard deviation

^b^ Commercial or public insurance
